# Where There Is Fire There Is SMOKE: A Scalable Edge Computing Framework for Early Fire Detection

**DOI:** 10.3390/s19030639

**Published:** 2019-02-03

**Authors:** Marios Avgeris, Dimitrios Spatharakis, Dimitrios Dechouniotis, Nikos Kalatzis, Ioanna Roussaki, Symeon Papavassiliou

**Affiliations:** School of Electrical and Computer Engineering, National Technical University of Athens—NTUA, GR 157 80 Zografou, Greece; dspatharakis@netmode.ntua.gr (D.S.); ddechou@netmode.ntua.gr (D.D.); nikosk@cn.ntua.gr (N.K.); ioanna.roussaki@cn.ntua.gr (I.R.); papavass@mail.ntua.gr (S.P.)

**Keywords:** IoT nodes, edge computing, control theory, resource scaling, social media, cyber-physical social system, fire detection

## Abstract

A Cyber-Physical Social System (CPSS) tightly integrates computer systems with the physical world and human activities. In this article, a three-level CPSS for early fire detection is presented to assist public authorities to promptly identify and act on emergency situations. At the bottom level, the system’s architecture involves IoT nodes enabled with sensing and forest monitoring capabilities. Additionally, in this level, the crowd sensing paradigm is exploited to aggregate environmental information collected by end user devices present in the area of interest. Since the IoT nodes suffer from limited computational energy resources, an Edge Computing Infrastructure, at the middle level, facilitates the offloaded data processing regarding possible fire incidents. At the top level, a decision-making service deployed on Cloud nodes integrates data from various sources, including users’ information on social media, and evaluates the situation criticality. In our work, a dynamic resource scaling mechanism for the Edge Computing Infrastructure is designed to address the demanding Quality of Service (QoS) requirements of this IoT-enabled time and mission critical application. The experimental results indicate that the vertical and horizontal scaling on the Edge Computing layer is beneficial for both the performance and the energy consumption of the IoT nodes.

## 1. Introduction

Recent climate changes in Europe have had a direct affect on the weather conditions that dictate how the forest fires evolve and react. In fact, in the last few years, Mediterranean ecosystems in the European South and several Scandinavian countries in the North have been increasingly suffering from unexpected wildfires [1]. Recent tragic events in Greece, with the deadly wildfires near the capital, gave additional boost to this work. In the past, these incidents would mainly have an impact on the wild fauna and flora, however, the interaction of humans with wildfires has significantly changed during last decades; the expansion of urban areas near forests, called Urban-Wildland Interface, put human population and their assets at higher risk of wildfires than ever before [2]. Thus, the strategy of the fire management and the preparedness towards the continuously extended severe fire danger season must be updated and enhanced.

In the smart computing context, Cloud Computing the Internet of Things (IoT) and big data analytics are properly orchestrated to provide assisting applications for human daily activities such as education, health and transportation. These complex applications are either time- and mission-critical applications with stringent requirements. Dealing with natural disasters, like wildfires, is an interesting field for the development of such applications, since the early and precise detection of a forest fire is the most important step for in-time firefighting. Various IoT node arrangements, for example Wireless Sensor Networks (WSN) and, more recently, Unmanned Aerial Vehicle (UAV) clusters equipped with remote sensing capabilities, have enabled the detection of wildfires [3,4] and the automatic notification of the responsible public authorities. The ability to perceive their environment and react to its changes, perform basic data processing and exchange information, alongside the excellent scalability and the low capital and operational expenditures, makes IoT networks a reliable solution for autonomous monitoring of large forest areas. However, as these networks typically comprise of small battery powered devices performing multiple tasks, limited energy resources and the scarcity of computation capabilities for real-time processing are the most important disadvantages towards their wide adoption in the fire-fighting domain. Dealing with these shortcomings, Edge Computing [5] is a new service delivery paradigm that can provide rich computation resources near IoT portable devices. In the specific case of wildfires, a cluster of powerful servers is placed at the edge of the network and enables the offloading and processing of the IoT nodes’ computation-intensive tasks, e.g, image recognition, in order to reduce their energy consumption and achieve the application’s strict time constraints.

The proliferation of social media use by large population proportions along with recent advances in data sensing, collection, storage and analysis, supports the realization of the participatory data gathering paradigm, also known as social sensing. Data that are produced on social media services can act as an additional source of information valuable in various application domains, including the scope of disaster prevention, detection, control and assessment. For example, recent research efforts have evaluated the use of social media in relation to extreme weather incidents [6], earthquake events detection [7], and to estimate diseases spread such as influenza [8] and malaria [9]. These initial findings suggest that social media may provide a promising approach for detecting and mapping environmental hazards and climate-related impacts, however a robust methodology has yet to be defined and validated. In particular, within the scope of wild-fire detection, social media users, who happen to be in the proximity of a fire incident, can provide valuable information and testimony about the current situation and help to timely and accurately detect a wildfire [10]. The potential of this approach has been also confirmed by European Commission’s JRC initiative named Digital Earth Nervous System [11].

Generally, all the aforementioned technologies can be parts of a Cyber-Physical System (CPS), which integrates computing, networking and human resources. More specifically, in this article, we propose a three-level Cyber-Physical Social System (CPSS) for early fire detection. At the bottom level, a network consisting of static or mobile IoT nodes monitors forest areas for detecting fires at their initial stage. Following the machine learning trend [12], these nodes are equipped with embedded camera modules to enable computer vision-based fire detection. Thus, at the middle level, a Scalable edge coMputing framewOrK for early firE detection (SMOKE), hosts two image classification services, which processes field snapshots captured from the IoT nodes. SMOKE is actually a dynamic resource scaling mechanism for IoT-enabled, time- and mission-critical applications, meant to be deployed at a cluster of servers at the network edge, in the nodes’ proximity, assisting the offloading of computationally intensive, energy hungry tasks. At the top level, a Cloud-based decision-making service combines the classification results of the previous level, users’ actions on the social media and other services, such as weather information services, in order to accurately infer the fire incident severity and notify the responsible authorities.

This study extends the work in [13] and the main contributions of it can be summarized to the following three main topics:

1. a vertical scaling mechanism; contrary to a Cloud Computing environment, the computational resources available on the servers located at the edge are limited [14]. Hence, the simultaneous tenancy of more than one applications at each Edge Server may risk the Quality of Service (QoS) satisfaction. Consequently, a dynamic resource allocation and admission control mechanism is developed with the use of a linear switching system and a state feedback controller.
2. a horizontal scaling mechanism; in the same direction, this optimization mechanism is responsible for the activation/deactivation of each Edge Server, the placement of the applications’ instances within them and the distribution of the incoming offloaded requests among those instances, while taking into account various performance criteria.
3. a Cloud decision making service; Among the main challenges in early detection of fire related emergency situations is the richness of the data that are gathered from various sources (either sensors or humans), the efficient and fast processing of them and finally the estimation of the criticality level of the emergency situation. In this work, the decision support system aims to combine data from diverse sources such as IoT-generated images, satellite information, historic weather data and social media services but at the same time aims to produce decisions in a timely manner.

The rest of the article is organized as follows. In Section 2, the current state of the art is presented. Section 3 demonstrates the architecture of the proposed CPSS, while in Section 4 the theoretical background and mathematical justification behind the developed components of the architecture is thoroughly presented and analyzed. The evaluation of the proposed system in an emulated environment comes at Section 5, while, finally, Section 6 draws the conclusions and possible extensions of this study.

## 2. Related Work

In this section, we present the most interesting studies in bibliography from the perspectives of IoT, Edge and Cloud Computing, social media and image-based wildfire and/or emergency detection.

Zhang et al. [15] composed a study on the application of IoT infrastructures on the fire fighting industries. In that work, the status quo regarding the usage and the main characteristics that make IoT devices appealing, was presented, while, subsequently, suggestions on the wider adoption of them in the fire fighting domain, were discussed, with China as the main example. In the same direction, the authors in [16] analyzed the trend of leveraging Cloud Computing and IoT techniques on agriculture and forestry. On the first part, several relevant applications of these paradigms were listed; there, forest monitoring for fire prevention held a significant place. On the second part, ideas on the combination of those two for maximizing vegetation benefits were proposed. Another IoT setting, this time in the form of a Wireless Sensor Network was studied in [17], to support early fire detecting activities; this work briefly discussed some indoors as well as forest based installations. Finally, on their search for additional flexibility, the authors in [18] used images captured from UAVs to detect forest fires. The Forest Fire Detection Index was utilised, alongside other classification methods for vegetation classification and tonalities of flames and smoke in order to assess the spread rate.

One of the difficulties that software systems aiming to integrate multiple information sources has to face is the homogenization of data. This issue, also known as data interoperability, is one of the key requirements in building cross-domain IoT applications and it gets even more complicated when the goal is to combine information sources from completely diverse software services areas (e.g., IoT and social networking services). There are various ongoing standardization efforts toward this scope and organizations such as IEEE Standards Association, AIOTI, oneM2M and W3C are in collaboration trying to reach consensus on defining common APIs and data models within the IoT application domain [19]. In addition, and with regards to Machine-to-Machine interoperability the European Telecommunications Standards Institute (ETSI) contributes to the worldwide standardization efforts along with OneM2M through the *oneM2M Global Initiative* in order to standardize a common M2M service layer platform for globally applicable and access-independent M2M services [20]. In the study [21], authors defined a common approach and data model able to represent in a uniform way information both from IoT environment and social media services. The data homogenization issue in the presented approach is tackled based on the adaptation of semantic and syntactic interoperability mechanisms which are detailed presented in [22,23].

In the case of wildfires, social media can be a powerful crowd-sensing tool for situation awareness and fast data diffusion. A review on the us of social media on forest fire detection was presented in [24]. This study categorized the wildfire risk management systems and the social media methodologies followed, crowd-sourcing applications developed and social media frameworks deployed for disaster management. Furthermore, a sensing process based on social media data management and a general architecture of a wildfire social sensor management platform were proposed. The following social media-based studies are the most relevant to our approach. Wang et al. [25] provided a Twitter-based spatial, temporal and content analysis for wildfires. The Kernel Density Estimation (KDE) method was used to analyze the possible spatial patterns of the tweets referring to the wildfires. This analysis was also combined with the temporal evolution of the tweets and a term frequency analysis to validate the ability of social media to characterize an emergency over time and space. Also, other parameters, such as the influence of the opinion leaders, were taken into account. Twitcident [26] was a web-based system connected to emergency broadcasting services that automatically searched, filtered and classified emergency situations. Additionally, analytical tools and users were allowed to make customized searches for specific events, including wildfires.

In the 5G context, dynamic scaling of Edge and Cloud Computing resources, i.e., the on-runtime, on-demand provisioning of the amount and type of server resources, plays a key role for the performance guarantee of time- and mission-critical applications. On the contrary, a priori, static resource provisioning fails to deal with unanticipated changes in resource demands [27]. Resource scaling can also be classified as either *Vertical* or *Horizontal*. With the term *Vertical Scaling*, we refer to the ability of increasing/decreasing the capacity of existing Virtual Machines (VMs) or containers by dynamically adding/removing CPU cores, RAM or storage; on the other hand, *Horizontal Scaling* deals with the activation/deactivation of servers and the number of virtual machines or containers to be placed in them. The interested reader can refer to [28] for a complete survey on Cloud elasticity. Leontiou et al. [29] proposed a hierarchical vertical and horizontal scaling framework for Cloud services. At the bottom level, fuzzy Takagi-Sugeno systems were used to model the dynamic operation of the VMs and a robust controller was designed to guarantee the Quality of Service (QoS) requirements and a stability analysis was discussed. At the top level, an unbounded knapsack problem was solved in order to simultaneously tackle the application placement within the active servers and the load balancing of the incoming requests into the VMs. Saikrishna et al. [30] proposed an algorithm to develop a multi-objective switching controller that ensured asymptotic stability with pole placement and addressed the problem of performance management of a web-server hosted on a private Cloud. Moreover, in [31], Grimaldi et al. used a PID gain scheduler to horizontally scale the available resources dynamically and achieve a desired CPU use. Similar to our work, the authors tried to maintain the control error close to zero by splitting the operating spectrum of each VM to distinct regions and solving an optimization problem to calculate the controller gains within them. Finally, the authors of [32] used operating regions, as well, and designed specific models to represent the behavior of each one of them; multiple fixed PI feedback controllers which alternated on runtime based on the operating region, comprised a switching control system that dynamically allocated CPU capacity to the VMs in order to achieve a desired average response time.

Contrary to Cloud Computing, little attention has been given to the dynamic resource scaling in Edge Computing settings. The resource provisioning problem on Edge Computing is usually dictated by the computation offloading strategy. *Computation Offloading* is the process of redirecting the heavy processing tasks of mobile or IoT devices to a nearby Edge Computing infrastructure for execution. Most of the proposed computation offloading studies used fixed modeling and static resource provisioning for the Edge Computing resources. However, these resources are, as we mentioned earlier, limited and a dynamic resource scaling approach is necessary to guarantee QoS. Jia et al. [33] proposed a load balancing framework for geographically spread cloudlets, i.e., small-scale data centers or clusters of computers designed to quickly provide Cloud Computing services to mobile and IoT devices, within close geographical proximity. The operation of each cloudlet was modeled with the use of queuing models and static provisioning of cloudlet resources was adopted. A scalable load balancing algorithm was then used to minimize the maximum average application response time of the cloudlets. In [34], the authors presented a comprehensive analysis on energy consumption modeling in Edge Computing settings. These models were classified as either static, flow-based or time-based. Furthermore, considering various network parameters, the energy consumption in Cloud and Edge Computing related scenarios was discussed. The experimental results demonstrated that computation offloading can significantly reduce the energy consumption of IoT devices. MAGA [35] proposed a mobility-aware, genetic algorithm-based decision system that aimed to improve the offloading success rate and reduce the energy consumption on mobile devices while the response time requirements were met. Frequent user mobility patterns were inferred via a tail matching sub-sequence mobile access prediction method and a modified genetic algorithm decided which components of the work flow were to be offloaded or executed locally otherwise. The resource provisioning of the cloudlets was considered static.

This article proposes a hierarchical CPSS that leverages the advantages of some of the abovementioned technologies. At the bottom level, the sensing capabilities of IoT devices are exploited for continuous fire monitoring. The computation intensive image processing is offloaded to the middle level, where the SMOKE framework implements the horizontal and vertical scaling of the Edge Servers’ resources, in order to guarantee specific response time requirements. Finally, at the top, Cloud Computing layer, the image classification results are forwarded and combined with various sensor data, as well as with a spatial and temporal analysis of social media actions and then a decision making service infers additional information on the incident severity. This information is subsequently forwarded to the responsible local authorities for further actions.

## 3. Requirements and System Architecture

The hypothetical scenario addressed in this work describes an IoT network, consisting of IoT nodes equipped with camera modules (e.g., Raspberry Pis or IQ FireWatch [36]), capturing images in order to detect emergency incidents (i.e., fire). At the same time, we assume that wireless sensors are scattered in the same forest area; in our case, a wireless sensor is nothing more than a low cost sensor, monitoring gas-emissions, humidity or smoke in the trees or vegetation. The proposed CPSS is envisioned as a semi-rural area installation were forests are in the proximity of a populated area. This has a twofold effect on the system; first, its whole operation is based on a local private network (e.g., WLAN) and a cellular network is not necessary. Second, civilians with mobile devices are present. The sensors’ data alongside the information provided from the IoT nodes and the social media traffic produced by the human factor, are fed to a decision-making service deployed on the Cloud, able to assert the risk level and give further guidance to public authorities. Finally, an *emergency mode* of the CPSS is specified, when, in the case of a possible fire outbreak and in order to better examine the incident, there is a rapid increase of the pictures needed to be analyzed.

Thus, regarding this IoT-based fire detection scenario, the following identified use-case requirements evince the importance of a scalable Edge Computing architecture to accommodate the offloading of the computationally demanding processes to the network edge.

- Timely incident detection and identification: The ability of wildfires to spread out extremely quickly [37], makes the detection and suppression at an early stage a necessity. Such time-critical applications, demand low-latency access to servers at the edge of the network, ability to perform rapid computations and take immediate decisions.
- Optimal use of IoT nodes resources: As mentioned above, although IoT nodes demonstrate excellent fire-detecting fitting capabilities like automation and control of their functionality in relation to their perception of the environment, wireless data transferring, small size and the ability to form scalable networks, they usually lack the computational and energy resources to perform complex tasks and operate autonomously for prolonged periods of time. As a result, frequent usage of sensors, communication and data processing has to be minimized in order to find a balance between increasing battery life and accurate incident detection. The proposed Edge Computing approach enables IoT nodes to offload energy and/or computational hungry tasks (i.e., image recognition) to servers in proximity, wirelessly via a local network. This placement enables low-latency access to the servers, contrary to the access to the remote Cloud through the public Internet, which might be unpredictable when it comes to response times.
- Ability to handle the application’s rapid scalability needs: The hypothesis that IoT nodes produce a fluctuating workload, depending on whether they operate normally or in the *emergency mode*, increases and decreases the offloading rate for a specific Edge Server rapidly. As a result, computational needs at the edge of the network may vary differently from time to time for the image recognition application. There is also the possibility that additional applications are co-located at the Edge Servers; this makes static resource provisioning a problematic situation that may lead to resource under-use and subsequently to hold the ability of applications to coexist at the same server back, or resource over-use which will possibly introduce delay to the execution off the offloaded requests and jeopardize the application’s mission-critical aspect. Thus, the need for fine grained resource allocation and QoS guarantee is evident.
- Interoperability of sensors’ Data: A critical obstacle when integrating information from heterogeneous sources is that the underlying information systems (e.g., IoT platforms) are mainly isolated and act as “vertical silos”. The lack of interoperability among these systems impedes the creation of cross-domain, cross-platform and cross-organizational services. To overcome these obstacles syntactic and semantic interoperability solutions are necessary to be enforced. To this end, syntactic interoperability is associated with the ability of systems to exchange information in order to communicate on a technical abstraction level. Semantic Interoperability, denotes the ability of different applications and business entities to understand exchanged data in a similar way, implying a precise and unambiguous meaning of the exchanged information.
- Privacy protection of individuals: A common challenge that society has to address in the recent years is to keep a balance between preventing and mediating the damage that occurs from disastrous situations without on the same time violating human rights such as the right of privacy protection of individuals. Advances on sensing technologies and data collection mechanisms make feasible the deployment of vast sensor networks that can potentially become intrusive and violate established regulations (e.g., GDPR). Edge computing paradigm assists in keeping the processing at the edge of the network thus avoiding the indiscriminate transmission and storage of sensitive information, such as image and video recordings.

**Figure 1:**
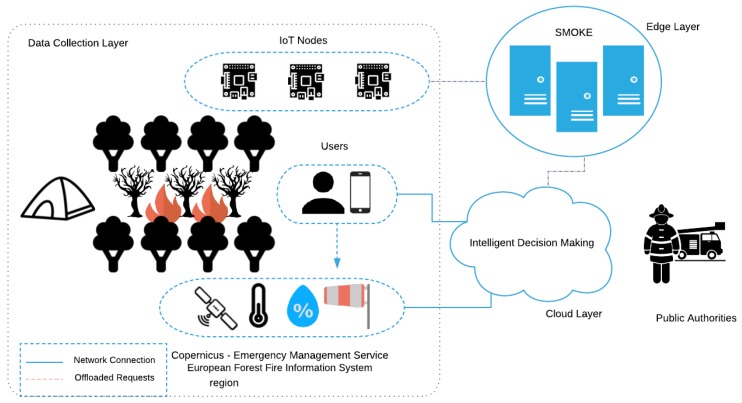
CPSS Architecture.

In this section we describe in more detail the design of our CPSS’ architecture. As depicted in Figure 1, the designed system consists of two main agents, namely the SMOKE framework and the Intelligent Decision Making component, and four subordinate agents which interact with each other and contribute in a unique way to deal with the emergency incident; the IoT nodes, the Sensors, the Social Media and the Public Authorities. The Intelligent Decision Making agent operates as a Cloud component gathering data from the other components, operating as the top layer of this CPSS. Although this proposed architecture is intrinsically linked with the early-fire detection use case, which is studied in this work, it can be easily adapted to accommodate a variety of settings with similar requirements.

### 3.1. SMOKE

The SMOKE framework follows a top-down design in a manner that there exists a centralized controller that makes the decisions which are in turn propagated to the lower levels of the architecture to be realized by local controllers. The proposed architecture is generally applicable in a single-site Edge Computing infrastructure but can be easily expanded for Edge-to-Cloud or Edge-to-Edge collaboration; its components are described in detail bellow.

#### 3.1.1. Containerized Applications

SMOKE supports the simultaneous management of co-hosted applications that are able to receive offloaded requests on the Edge Servers. The only prerequisite is that those applications be containerized. In this work, for the sake of demonstrating the multitenancy efficiency of SMOKE, we developed two TensorFlow-based object recognition applications able to recognize images containing events of interest (i.e., fire), which were trained off-line in a supervised manner. These applications were then containerized and deployed on the Docker Platform installed of each Edge Server. Containers were selected instead of VMs, as a means of virtualization, because of their overall lower overhead, smaller footprint and lightweight vertical scalability.

#### 3.1.2. Central Controller

**Figure 2:**
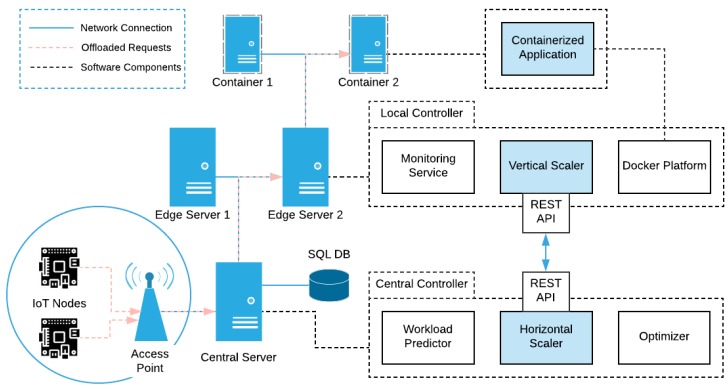
SMOKE Architecture.

The offloaded traffic, generated by the IoT nodes, is directed to the Central Controller (located on the Central Server) through a local Wireless Access Point. Here lies the upper level control process of our mechanism, as depicted in Figure 2. To accommodate this control process, time in our framework is quantized in discrete time intervals; at the beginning of each time interval this component selects an appropriate container formation to be implemented to each Edge Server directly connected to it and consequently distributes the incoming workload accordingly. This formation defines the number of active servers alongside the number and the operating point of the containers to be placed in them. With the term *operating point* we refer to the number of the offloaded requests that each container will accept, the number of cores that it will be allowed to use, as well as the average response time it is requested to achieve during the next time interval. These operating points of the containers are calculated on the *Local Controllers*, as described in the next section. This control process, hereinafter referred to as *Horizontal Scaling*, is performed in an on-line and proactive manner, leveraging an internal prediction mechanism, the *Workload Predictor*, which provides an estimation on the number of requests to be expected on the time interval, for each application. The essential input for this estimation process is provided by the Monitoring Service component of the Local Controller deployed in each Edge Server, which is responsible for collecting data regarding both the network traffic (i.e., offloading requests admitted and end-to-end response times) and the containers’ resources use (i.e., CPU usage) at each given time. Then, the *Optimizer* component uses the output of the Workload Predictor and the feasible operating points, calculated offline, and computes the optimal number of active servers and containers required to meet the different performance criteria. The theoretical background of this process is discussed in more detail in Section 4. Hence, depending on the aforementioned decision and considering the predicted workload for each time interval, the Central Controller dictates the creation, scaling and destruction of the application-specific containers to the Local Controllers accordingly. Also, at the end of each time interval, the average classification score of the offloaded images is calculated here. Additionally, when a classification score above a predefined threshold emerges, indicating a possible fire outbreak, information is transmitted to the respective IoT node regarding its new operating mode (normal or emergency). The time between the capturing of the image of interest and the transmission of the updated IoT node information is defined as the application’s response time. All the monitoring data involved in this process are stored in a relational database present in the Central Server, in order to be used for demonstration purposes. It is also worth noting here that, physically, the Central Server is nothing more than an Edge Server, located on the users’ proximity, with a more advanced role; this of the decision maker.

#### 3.1.3. Local Controller

At the lower level, each Edge Server is equipped with a Local Controller, responsible for both gathering the request-related statistics from the containers, we mentioned earlier, needed for the Monitoring Service and tackling the small fluctuations of the incoming workload, according to the predicted number of requests for each time interval. The lower level control process implemented in this component moderately scales the containers vertically, providing the required resources, thus realizing the decision made by the Central Controller. In more technical terms, the communication between the Central Controller and the Local Controllers is performed via a REST-API present in the latter; the Local Controller also uses the *Docker Platform* in order to scale the containers. This *Vertical Scaling* ensures that the containers remain within and area around the selected operating point, hence guaranteeing minimum and stable application response times, in order to meet certain QoS requirements. Section 4 provides further mathematical justification for this process.

### 3.2. Intelligent Decision Making

This Intelligent Decision Making (IDM) service is deployed on the Cloud Layer of the proposed architecture. The geo-tagged images are processed and classified at the SMOKE framework on the Edge Layer and, if the average classification score is above a confidence threshold of fire and smoke detection, then an ongoing emergency situation probably occurs and the IDM’s operation is triggered. In order to further evaluate the criticality of the incident at the targeted area additional data are collected by the *Data Collection Engine* in order to be fed to the *Decision Algorithm*. The latter applies logical rules on the provided data collection in order to timely infer the level of the associated danger of the situation and render the respective estimation in an intuitive manner.

#### 3.2.1. Data Collection Engine

**Figure 3:**
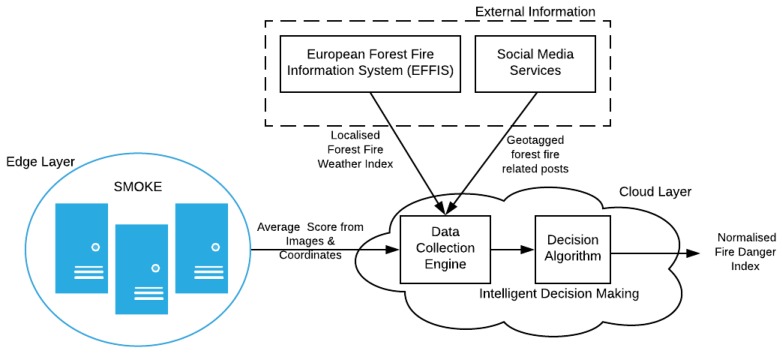
Intelligent Decision Making Architecture.

As it is illustrated in Figure 3, the main data sources integrated with this system are:

- SMOKE service: The SMOKE service provides the classification score of the images along with the time and location, in terms of GPS coordinates corresponding to the IoT node’s location, from the associated area that the images was captured. It is the image classification score that triggers the overall data gathering process, while additional data are retrieved based on location criteria indicated by the respective images coordinates. To this end, a reverse geo-coding process is applied in order to extract the associated readable addresses and place names which are particularly useful in the retrieval of data from social media services.
- European Forest Fire Information System: The European Forest Fire Information System (EFFIS) calculates on a daily bases an index, called Forest Fire Weather Index, for all the regions of EU based on environmental and weather related information, such as the humidity of the air at the beginning of the afternoon; the temperature in the middle of the afternoon; the precipitation during the last 24 h; the maximum speed of the average wind. The fire danger is mapped in five classes with a spatial resolution of about 16 km. The fire danger classes are the same for all EU countries and information is provided encoded as GeoTIFF format maps showing a harmonized picture of the spatial distribution of fire danger level throughout EU. The GeoTIFF standard allows georeferencing information to be embedded within a TIFF file. The Fire Danger Forecast maps are updated daily and are freely available from the EFFIS online service [38]. The actual file has a small size (<1 MB) hence it can be efficiently processed. The five classes of the Forest Fire Weather Index embedded as different color codes (bands) in the GeoTIFF file are:
  1. **LOW**—Fuels do not ignite readily from small firebrands although a more intense heat source, such as lightning, may start fires in duff or light fuels.
  2. **MODERATE**—Fires can start from most accidental causes, but with the exception of lightning fires in some areas, the number of starts is generally low.
  3. **HIGH**—All fine dead fuels ignite readily and fires start easily from most causes.
  4. **VERY HIGH**—Fires start easily from all causes and, immediately after ignition, spread rapidly and increase quickly in intensity.
  5. **EXTREME**—Fires start quickly, spread furiously, and burnt intensely. All fires are potentially serious.
- Social networking services. In the current implementation of the IDM the Twitter micro-blogging platform has been integrated. Twitter maintains a total number of 335 million monthly active users, who produce more than 500 million number of Tweets per day. The fact that 80 percent of Twitter users use the service through mobile devices, makes this social network an ideal platform for applying the social sensing paradigm. In addition, Twitter has been selected in the scope of the work presented in this article due to its openness and the almost unrestricted access to the publicly available user provided content and profile information through APIs. Data collection for the needs of the IDM is facilitated through hashtags and keywords associated with wildfires combined with tags denoting geo-reference. Although Twitter offers the option to geo-tag the provided Tweets, this feature is not frequently used, thus it can not be exploited effectively for the needs of the IDM service. On the other hand, it is a common practice for Twitter users to introduce their own tags in order to express the connection of their post with an area. The reverse geo-coding allows the IDM to extract a set of local area names, also expressed in local language, which will be used as keyword criteria for retrieving Tweets that potentially are associated with an emerging wildfire incident.

#### 3.2.2. Decision Algorithm

The Decision Algorithm aggregates information from the described sources, homogenizes their input and generates a normalized score that ranges from zero to one (highest value) denoting the emergency level. The overall operation is triggered periodically by SMOKE classification score and runs continuously, meaning that the IDM may receive multiple images’ scores from various locations, where a decision should be extracted for each case. As it is already stated, the image classification score is the main criterion for the early identification of the fire incident while the additional data gathered are utilised in order to further evaluate the severity of the incident in terms of human presence in the area, potential fire spreading, etc.

## 4. System and Process Modeling

### 4.1. SMOKE Scaling Framework

As described in the previous section, a single SMOKE deployment consists of the many Local Controllers and one Central Controller; each Local Controller aims at controlling and regulating the operation of the containers that run on the same Edge Server with it; the Central Controller makes the decisions on the activation of the Edge Servers and the respective containers, as well as the load balancing of the incoming requests. Trying to be compliant with the taxonomy defined on the survey on [28], the Local Controller was designed to use linear switching systems for modeling the containerized applications and a state feedback controller for each linear system, designed to apply admission control decisions. At the same time, the Central Controller solves a mixed integer programming problem to determine the number of active servers and containers, which are necessary for serving the total workload of the hosted applications.

**Local Controller:** As mentioned above, the dynamic operation of the containerized applications is modeled with the use of switching linear systems, with the switching criterion being the number of the allocated CPU cores to each container. This modeling approach captures the dynamic behavior of the containers under different operational conditions and enables us to perform indirect resource allocation. In our case, the various operational conditions, under which the modeling is performed, include different image sizes, resolutions and transmission delays (depending on the network congestion at each given moment) per request and a variety of request rate values. So, for each different CPU core allocation, the operation of the container is described by a discrete linear system of the following form,

(1) x(t+1)=ax(t)+bu(t),

where x(t) is the *state variable* that expresses the average application response time for time interval *t* and u(t) is the *control variable* that represents the number of the admitted requests within time interval *t*. Here, with the term admitted we describe the requests that are actually allowed to the container for processing. The parameters α and *b* of the above model are estimated by using the Recursive Least Square (RLS) algorithm [39].

Physically, a container with *c* allocated cores is constrained to serving up to $u_{e}$ requests of the containerized application while maintaining an average response time of $x_{e}$. This pair $\left(x_{e},u_{e}\right)$ is called an operating point and generally, for each such switching system, a set of feasible operating points of this kind can be computed according to various performance criteria and while taking into account the constraints of the state and input variables. In our case, these feasible operating points are computed by solving the following linear programming with the goal of maximizing the number of the admitted requests:

(2) $$\begin{array}{c} \max_{x_{m},x_{M},x_{e},u_{m},u_{M}}\;u_{e}\\ \mathrm{subject}\;\mathrm{to}\\ x_{e}=ax_{e}+bu_{e}\\ x_{m}\leq x_{e}\leq x_{M}\\ u_{m}\leq u_{e}\leq u_{M}. \end{array}$$

The first constraint implies that each operating point must also be an equilibrium of the discrete linear system and this will guarantee its stability and confinement in a specific operating area around it. The second constraint dictates that the state variable must lay between a minimum $\left(x_{m}\right)$ and a maximum value $\left(x_{M}\right)$ set by the application’s requirements, while the last constraint refers to the control variable varying between the minimum available $\left(u_{m}\right)$ and the maximum available value $\left(u_{M}\right)$.

**Figure 4:**
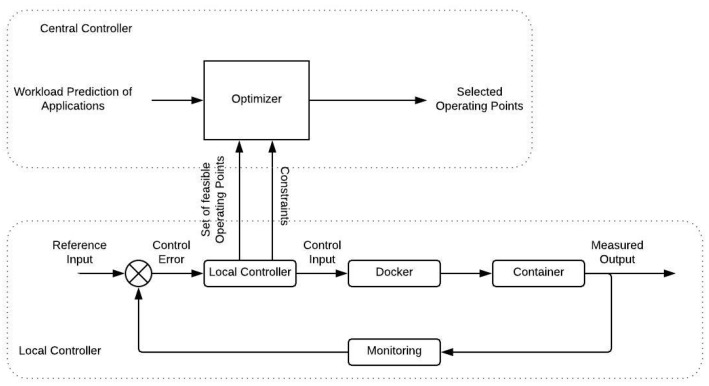
Feedback control system in Vertical Scaling.

For each operating point of every linear system, a state feedback controller is designed in order for the respective containerized application to meet the response time requirements. This control law is defined as,

(3) $$u\left(t\right)=K\left(x\left(t\right)-x_{e}\right)+u_{e},$$

where K∈R is the control gain. Applying (3) in (1), we get the closed loop form of the linear system,

(4) x(t+1)=(a+bK)x(t)+c,

where $c=b(u_{e}-Kx_{e})$ is a constant term. Regulating the eigenvalue λ=a+bK of the system (4), the stability of the closed loop system and the convergence speed to the equilibrium point are affected. Thus, we select a stable eigenvalue, which lays inside the unitary circle, in order to compute the control gain $K=\frac{\lambda -a}{b}$. To give a better understanding of the whole process Figure 4 illustrates a block diagram describing the closed loop system. The following list explains the role of each signal presented there,

- *Reference Input*: The average application response time for the respective operating point $x_{e}$.
- *Control error*: The difference between the actual average response time of the last interval and the reference value, $x-x_{e}$.
- *Controller*: An affine switched state feedback control process, as the main process in the Local Controller.
- *Control Input*: The maximum request rate to be admitted at the container for the next time interval, computed by (3).
- *Docker*: The Docker Platform as the the control system’s actuator.
- *Container*: The containerized applications as the controlled process.
- *Measured Output*: The measured average application response time of the container for the previous time interval.
- *Feedback*: The sensor of the control system, monitoring and recording its current state at each time.

**Central Controller:** The main functionality of the Central Controller on the top layer, is to decide the switching action for the Horizontal Scaling process, as described in Section 3.1.2. As shown in the upper part of Figure 4, the Optimizer component consumes information regarding the available operating points and the predicted workload, for the next time interval, for each application. The prediction of incoming workload, estimated according to a linear trend forecasting procedure as described, in [40], alongside the operating points facilitate the formulation of an optimization problem solved by the Optimizer; as mentioned earlier, the assumption that each application is deployed to at most one container per Edge Server is made. Furthermore, at a preliminary stage, an offline exhaustive procedure where all the feasible combinations of the containerized applications’ operating points within a server, is calculated. Feasible combinations are the ones that do not exceed the total computational capacity of the respective server. Then, the Optimizer minimizes the number of active servers and the allocated CPU resources by solving, sequentially, two mixed integer linear optimization problems (MILPs). At the first MILP, the minimum number of Edge Servers to be activated on the next time interval, in order to serve the estimated workload, is determined by solving the following,

(5) $$\begin{array}{c} \min_{S,P,A,{\tilde{R}}_{i}}\;S_{a}\\ \mathrm{subject}\;\mathrm{to}\\ S_{a}\in S\\ p_{ij}\in P\\ p_{ij}\in \left[0,S\right]\\ \sum_{\forall i\in A}\sum_{\forall j\in S}p_{ij}u_{ei}\leq {\tilde{R}}_{i}. \end{array}$$

Here, the first constraint dictates that the servers to be activated are selected from the pool of the available servers. The next two constraints imply that each of the selected containers to be deployed on the selected servers corresponds to an existing operating point and that their placement on the active servers must correspond to an acceptable combination. Finally, the last constraint means that the estimated total workload for each application, ${\tilde{R}}_{i}$, must be served by the containers. On the second MILP, the minimum amount of computational resources, in terms of CPU cores per container per server, are computed, while taking into account the result of the previous optimization,

(6) $$\begin{array}{c} \min_{S_{a},P,A,{\tilde{R}}_{i}}\;\sum_{\forall i\in A}\sum_{\forall j\in S_{a}}p_{ij}C_{ij}\\ \mathrm{subject}\;\mathrm{to}\\ p_{ij}\in P\\ p_{ij}\in \left[0,S\right]\\ \sum_{\forall i\in A}\sum_{\forall j\in S}p_{ij}u_{ei}\leq {\tilde{R}}_{i}, \end{array}$$

where $C_{ij}$ is the number of allocated CPU cores to the container of the *i*th containerized application on the *j*th activated server. The reason behind distinguishing the optimization process into two distinct subproblems is that, at first, we tried to solve a Multi-Objective MILP by combining the two optimization targets to one weighted objective function, but that led to the one objective being successfully optimized while the other one concluded to a sub-optimal solution, regardless of the assigned weights. Also it should be noted that the number of Edge Servers is relatively small so the overall computation time of these optimization processes does not disrupt the Central Controller’s smooth operation.

### 4.2. Decision Making Algorithm

As already stated, the decision making process is triggered by the SMOKE system when the fire-related average classification score of images, captured by the IoT node, is above a predefined threshold, thus indicating a considerable probability of fire occurrence. The respective score sent to the IDM is denoted by $S_{im}\left(t,p\right)$, where *t* is the time when the image was captured by the IoT node and p=(x,y) expresses the GPS coordinates of the point where the IoT node was located at upon image capturing (latitude *x* and longitude *y*). As already stated, the value of $S_{im}\left(t,p\right)$ lies within the range of [0,1].

Once $S_{im}\left(t,p\right)$ is obtained, the IDM triggers a reverse geo-coding process in order to map the GPS coordinates to a specific country and language, and to extract multiple area names for the specified point. The output of this process is a set of location-related keywords or tags denoted by $L\left(p\right)=\left[l_{1}^{p},l_{2}^{p},\ldots ,l_{N}^{p}\right]$. Various combinations $C_{r}^{lf}\left(p\right)=\left[l_{i}^{p},l_{j}^{p},\ldots ,f_{k},f_{l},\ldots \right]$ of these tags coupled with terms $f_{i}$ linked with fire emergency are generated and are subsequently fed to the Twitter API in order to fetch all Tweets that involve the specific term combinations and are posted in the latest window frame of duration Δ. The population $N_{r}^{lf}$ of the retrieved Tweets $TW_{r}^{lf}\left(C_{r}^{lf}\left(p\right),\left[t-\Delta ,t\right]\right)=\left[tw_{r1}^{lf},tw_{r2}^{lf},\ldots ,tw_{rN}^{lf}\right]$ that use both keywords linked to the area of interest as well as terms related to fire emergencies is then averaged by the population $N_{r}^{l}$ of all Tweets $TW_{r}^{l}\left(C_{r}^{l}\left(p\right),\left[t-\Delta ,t\right]\right)=\left[tw_{r1}^{l},tw_{r2}^{l},\ldots ,tw_{rN}^{l}\right]$ that only use keywords linked to the area of interest. A respective social media score $S_{sm}\left(t,p\right)=\frac{\sum_{r}\frac{{N_{r}^{lf}\left(t,p\right)}^{2}}{N_{r}^{l}\left(t,p\right)}}{\sum_{r}N_{r}^{lf}\left(t,p\right)}$ is eventually calculated that lies within the range of [0,1].

With regards to the Fire Weather Index (FWI) provided by EFFIS, the IDM retrieves the respective values from the GeoTIFF image that correspond to the specified GPS coordinates of the point of interest p=(x,y). To deliver this, mapping of the GPS coordinates above to the GeoTIFF geo reference system is necessary. The value of the retrieved FWI for the specified coordinates is FWI(t,p) and the respective normalized score is $S_{FWI}\left(t,p\right)=\frac{FWI(t,p)}{5}$ that lies within the range of [0,1].

Finally, the IDM estimates an overall score $S_{fire}\left(t,p\right)$ of fire incident at point *p* and time *t* that indicates the severity of fire incident occurrence and is expressed as $S_{fire}\left(t,p\right)=w_{im}\cdot S_{im}\left(t,p\right)+w_{sm}\cdot S_{sm}\left(t,p\right)+w_{FWI}\cdot S_{FWI}\left(t,p\right)$ where the weights $w_{im}$, $w_{sm}$, $w_{FWI}$ indicate the significance of the respective scores, lie within the range of [0,1] and for which it stands that $w_{im}+w_{sm}+w_{FWI}=1$. The experiments conducted so far have assumed that $w_{im}\geq w_{sm}\geq w_{FWI}$, considering the reliability of the respective information sources. The nature of the assigned weights indicate that the image classification engine is considered as more important for the initial detection of the fire incident while the other two sources of information (social media data and EFFIS) have a complementary role in further evaluating the severity of the situation.

## 5. Experiments and Evaluation

In this section the experimental setup is thoroughly presented alongside an evaluation of the obtained results. At first, the significant role of SMOKE is evinced, after intense experimentation for proof of concept of the Horizontal and Vertical resource scaling on an Edge Computing topology and a comparison of the proposed architecture with the naive solution of the static resource allocation is presented. Subsequently, a time-related evaluation on the socially-aware intelligent decision making component, with input from social media, additional sensors’ data and image classification scores produced by the SMOKE framework, is discussed.

To demonstrate the operation of a SMOKE installation, the hypothetical setting of a forest and IoT nodes mounted on UAVs that fly over it, while capturing images in order to detect fire occurrence, is emulated. As discussed at Section 3, at the same time we assume the presence of wireless sensors and civilians using social media in the field. Furthermore, two additional assumptions are made; first, that due to the large space UAVs need to cover and investigate, each one of them takes over a smaller area, while, second, in the case of a possible fire outbreak, they are dictated to gather to the area of interest to better examine the incident, leading to a rapid increase of the pictures offloaded. UAVs were selected as the evaluation scenario because, although they pack amazing characteristics like operational versatility and durability in various weather conditions, they usually lack the resources to accommodate long-spanning missions, like vast forest areas surveillance. Additionally, the hypothesis that UAVs gather in a specific geographic area when certain events occur, and spread otherwise, allows us to denote the fine-grained, scaling-enabled resource allocation taking place on our system.

At this point, it should be made clear that UAV-based image analysis and fleet coordination algorithms are decoupled from the CPSS initial design and operation. Therefore, given the context and focus of this paper, UAV actual, real world operational methods and algorithms—though very challenging and of high practical importance—are considered out-of-scope of this study. They just serve the purpose of a conventional use case to enable the demonstration of the capabilities of such a combination of an Edge Computing framework and a decision-making platform.

### 5.1. SMOKE Evaluation

The first experiment illustrates the performance of the SMOKE framework when deployed on the NETMODE testbed [41] at the National Technical University of Athens in Greece. In this case, two identical 16-core Edge Servers with 16 GB of RAM were used, that each hosted two TensorFlow [42]-based applications, deployed in separate Docker containers as explained in Section 3. Without loss of generality, we made the assumption that each Edge Server can host exactly one container of each of these applications, due to the fact that more than one instances of the same service would introduce additional overhead costs when deployed on different containers on the same host. The differentiation between those two applications, developed solely for the experimentation purposes, was that the first implemented an image classification for conventional, visible light pictures, while the second one for infrared pictures. The model trained for the conventional image recognition was fed with a specific dataset [43] containing either pictures of forest wildfires or plain forests. For the infrared recognition model, we used a synthetic dataset, generated from the aforementioned one, by applying an infrared Photoshop effect to each image. Following the architecture described earlier, these Edge Servers also hosted an instance of the Local Controller. Regarding the emulation of the UAVs, two Raspberry Pi devices were acting as the mobile nodes, each of which was assigned to offloading requests to the Edge Servers, targeting one of the two applications. The mobile nodes were connected via Wi-Fi to an Alix3d2-based node hosting the Central Controller, which was in turn linked via Ethernet to the two identical Edge Servers. This whole setting represented a fully deployed SMOKE installation in a forest area. It should be once again noted that the process from the moment a UAV captured an image and offloaded it to the proximate Edge Server Infrastructure for processing until the result was calculated, as stated before, was defined as the response time. In addition to this, it was assumed that the Edge Servers were responsible for the UAVs’ operating mode, depending on the situation’s severity. Thus, the time until the detection of a possible fire incident, i.e., the response time, should kept below an acceptable value. Finally, the average classification score of the captured images was calculated and advertised to the IDM on the Cloud nodes, at each time interval.

**Figure 5:**
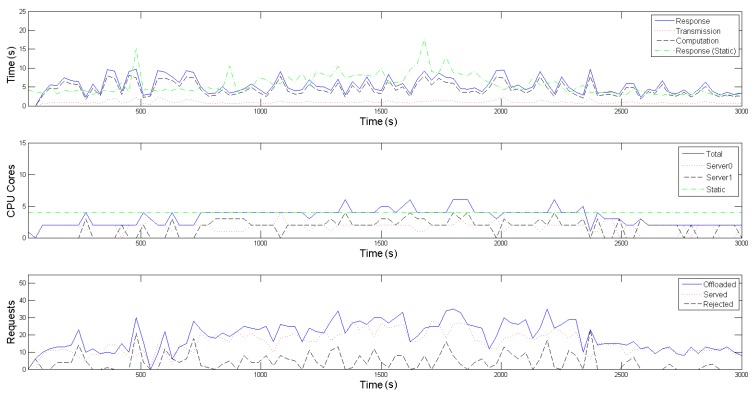
Conventional Tensorflow application.

**Figure 6:**
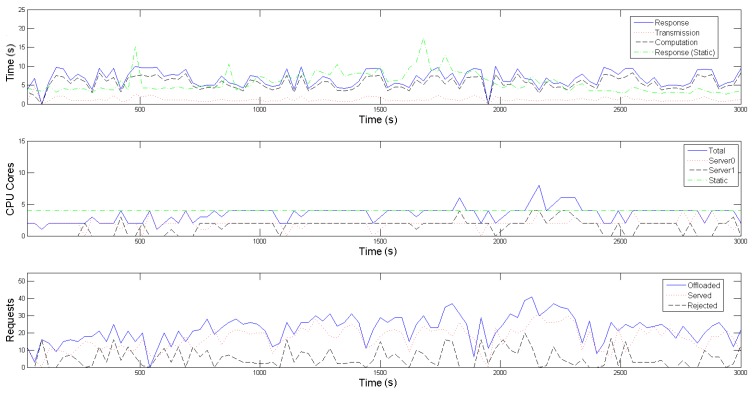
Infrared Tensorflow application.

In the presented emulation, this time interval, that additionally defines the overall operation of the SMOKE framework, as explained in Section 3, is set to 30 s. To make the offloading patterns more plausible, the amount of requests produced within the interval follows a Poisson distribution, while the inter-arrival time between two successive requests follows an exponential distribution. As depicted in the third diagrams of Figure 5 and Figure 6, the experiment scenario kicks off with the Raspberry Pis offloading an average of around 10 requests per time interval (blue-colored, solid line), for each application, simulating a normal period where no fire indications are present in the forest area (*tracking mode*). After approximately 5 min, the average number of requests per interval starts escalating, mimicking the UAVs’ behaviour of gradually approaching the area of interest, i.e., the framework’s proximity, when an emergency situation is detected *(emergency mode*). This average value peaks and stabilizes at 25 from the 20th to the 40th simulation min, where it is assumed that a fire has been recognized and more visual coverage of its area and spread rate is required. Finally, in the last part of the emulation, the offloading request generating rate returns to normal values, reflecting the wildfire being put under control and subsequently the UAVs reverting back to their normal operating mode.

Regarding the evaluation of the scaling mechanism of our framework, one can observe in the second diagrams of Figure 5 and Figure 6, that the Central Controller’s Horizontal Scaler adapts to the increased requirements in computational resources while on emergency mode (blue-colored solid line) and dictates the activation of a second server (black-colored, dashed line) between the 10th and 40th min of the emulation, in order to accommodate the mobile devices’ higher average offloading request rate. Contrarily, for the most of the initial as well as the final part of the emulation, only one server is active (red-colored, dotted line), proving to be adequate for the tracking mode of the UAVs. As explained thoroughly in Section 3 and Section 4, the Workload Predictor component estimates the incoming workload for the next time interval and then the Horizontal Scaler selects an appropriate formation, in terms of number and operating point of containers to be placed in the active servers, for each following time interval. Table 1 contains the offline calculated operating points for both application-specific containers, from which the Horizontal Scaler gets to choose; each operating point defines the nominal amount of offloaded requests, $u_{e}$, that the respective container is able to process, alongside the reference input $x_{e}$, when 1, 2, 3 or 4 Cores are allocated to it. A remark regarding the restriction of cores to be made available to each container to 4, is that this seemed to be a plateau where the containers became saturated and could not serve significantly more requests, despite allocating more cores to them. Furthermore, one can observe that certain outliers in the Poisson distribution, of either rapid increase or decrease in the offloading requests, cause the Workload Predictor to poorly estimate the incoming workload for the following time interval; this results to an increased amount of rejected requests, as well as a slight oscillation on the number of activated servers.

With respect to the Vertical Scaling part of SMOKE, the admission control process, executed on each Local Controller of each server, results in the rejection of approximately 19.58% of the offloaded requests for the conventional image recognition application and 23.01% for the infrared one (black-colored dashed line in each third diagram of Figure 5 and Figure 6). This is a consequence of the real incoming workload of the interval exceeding the projected one. Although the rejected volume is not negligible, it is not detrimental to the event detection precision. As a reminder, both these scaling processes aim to maintain the average response time below an acceptable value, Tref, which is an application-specific value. To achieve this, the reference input $x_{e}$ of each operating point is empirically set near to $\frac{Tref}{3}$. In our scenario, Tref is set equal to 10 s for both applications, thus the reference inputs of the operating points in Table 1 are set to 3 s and 3.5 s respectively. This goal is achieved, as depicted with the blue-colored solid line in the first diagrams in both Figure 5 and Figure 6; the response time remains within the limit of 10 s, despite the workload fluctuations. In these diagrams, average transmission and computation times are also plotted with red-colored, dotted and black-colored, dashed lines respectively; one can easily note that the average response time follows the same patterns as the average computational time, meaning that it is mainly affected by it. The average transmission time is negligible due to the use of the IEEE 802.11ac standard, which provides high throughput for the images (size of about 5 MB) used in this experiment. Finally, the vertical scaling of SMOKE is compared to a static allocation of four cores for each application; with the green-colored, dot dashed line on each first subfigure we denote the average response time of this static allocation, while on each second the total cores statically allocated. One can easily observe that when the experiment is on the tracking mode, it demonstrates better average response times for both applications, however, it still suffers from the phenomenon of overprovisioning; that is when a Container uses resources, that could be allocated other processes, without significant benefits. On the other hand, when the experiment enters the emergency mode, there are times when 4 statically allocated cores are inadequate for the processing requirements, resulting in violation of the 10 s limit for the average application response time. This problem of providing less than the necessary resources is called underprovisioning and it potentially puts the mission’s accuracy into risk.

### 5.2. IDM Evaluation

As stated in Section 4.2, the socially-aware Intelligent Decision Making process is triggered when the SMOKE component calculates an average classification score of the images offloaded in the last time interval that is associated with a high probability of fire incident detection. In this subsection, the evaluation results of the IDM component are presented focusing on the overall time overhead that is imposed until a final fire detection decision is reached. To this end, several experiments have been executed in order to identify the average time delays imposed by the various individual steps of the IDM algorithm. These steps are the following:

- Step A: Reverse geocoding process of the provided GPS coordinates, which is based on the call of external APIs (e.g., Google Maps API) in order to obtain a set of area names and location identifying keywords.
- Step B: Extraction of Fire Weather Index extracted from the GeoTIFF image based on the provided GPS coordinates.
- Step C: Collection and analysis of Twitter posts that are related with the indicated area.

**Figure 7:**
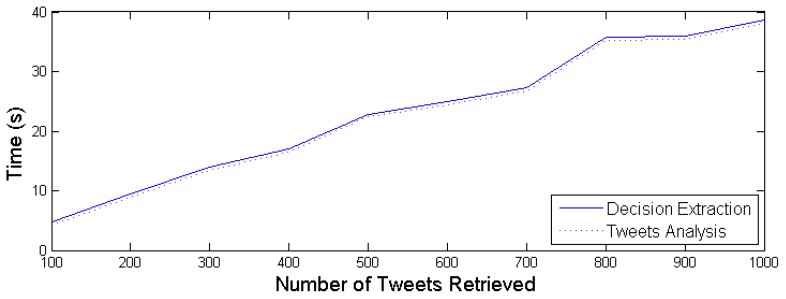
Decision extraction in relation to amount of Tweets retrieved.

After executing several experiments, the results obtained indicate that the average time required for Step A is 0.42 s, while the average time needed for Step B is 0.08 s. As one can easily observe, the introduced time overhead is minimal. On the other hand, Twitter data retrieval and Tweet processing may introduce a significant time overhead that depends heavily on the overall number of Tweets that comply with the retrieval criteria (e.g., Twitter posts containing tags and keywords related with the area and a forest-fire incident). Figure 7 illustrates the aggregated time needed for the IDM to perform steps A, B and C for increasing volumes of retrieved Twitter data. In order to estimate the expected volume of Twitter data that is generated during wild-fire incidents, a thorough review of existing approaches has been conducted and an analysis of the respective datasets indicating the evolution of the Tweet posts upon fire incident occurrence. The results, presented in Table 2, show that the total number of Tweets are not more than 10 K for the entire duration of the wild-fire incident. In our case, the Twitter stream API is utilised and the retrieval and processing of Tweets is taking place in an online mode aiming to detect the incident as soon as possible. Hence, it is safe to assume a few hundred Tweets to be generated within the first minutes of the wild-fire ignition. Therefore, given the results captured in Figure 7, the overall time overhead imposed by the social media mining process lies between 5 and 40 s, which enables the proposed IDM to reach a decision in a timely manner.

All in all, these two proposed CPSS mechanisms work seamlessly and efficiently towards the time- and mission-critical application of the early forest fire detection. The SMOKE framework manages to alleviate the IoT nodes’ computational workload, timely dictate their operating mode and orchestrate their new formation, should it be deemed necessary, while the servers’ resources are optimally used. Subsequently, the IDM is fed with the needed information that enables it to communicate the decision promptly to the public authorities.

## 6. Conclusions and Future Work

In this article, a Cyber-Physical Social System for early fire detection is presented; a Scalable Edge Computing Framework, called SMOKE, is deployed on the network Edge and is receiving captured images from several IoT nodes to evaluate the criticality of the situation; on a higher level, an intelligent decision-making service is deployed on the Cloud, receiving data from various sources (i.e., various low cost sensors, social media users and SMOKE) and communicates with the local authorities in case of emergency. Our work focused on two main aspects, namely (a) the Horizontal and Vertical scaling of the available Edge Servers’ resources, in order to achieve optimal allocation and use of resources and (b) the decision making mechanism, for a time-critical application, that takes the social factor into consideration. The proposed computation offloading mechanism is generic and applicable on several types of Mobile Edge Computing (MEC) environments and applications. The experiments conducted and presented in the previous section allowed us to draw some significant conclusions regarding the performance of the proposed frameworks. Horizontal and Vertical Scaling of Edge Servers is essential for guaranteeing the QoS metrics of time- and mission- critical applications, while dynamic resource allocation prevents over- or under- provisioning of the Edge Servers’ resources. Moreover, admission control on the incoming offloaded requests is a key factor for time-critical applications with stringent requirements in terms of retention of the desired QoS levels, like average task execution and transmission latency. Finally, the evaluation with regards to the time needed for the IDM component to collect the necessary data and extract a decision, shows that the overall time overhead is not higher than 40 s. This time duration is considered within the time limits of such a time-critical system especially considering the heterogeneity of the collected information types and sources. Having established a time-efficient data collection mechanism, among the authors next steps is the evaluation of the provided recommendations and their comparison with ground truth data.

Once again we find it significant to highlight the fact that the actual UAV operation is not a part of the CPSS’s core performance evaluation as it is decoupled from it. However, the extraordinary UAV capabilities could further assist demonstrating the essential scope of this work. Thus, for future work, a real-world experimentation of this proposed CPSS architecture will be in the spotlight. Potential infrastructures for this purpose include the federation of testbeds provided by H2020 RAWFIE [47] project, which aims at providing research facilities for IoT devices, including UAVs, and supporting experiments with monitoring tools and mechanisms for safely conducting realistic simulations. In this direction, RAWFIE provides AeroLoop [48], a simulation environment that allows users to conduct experiments with multiple virtual UAVs (vUAVs) in a flexible and controlled way and to thoroughly test and deeply discover the framework’s capabilities before trying out system-software and missions in the real world. Another issue that will concern us is the use of the LTE technology as an alternative to the WiFi access points currently used for the communication between the IoT nodes and the SMOKE framework. This will allow our framework to expand its operational range beyond semi-rural located forests and enable the coverage of vast areas. Hence, evaluating and conducting a comparative study on trying to minimize the data transmission overhead will be essential for a complete study in IoT-based early fire detection.

It should be also noted that, in this study, SMOKE is deployed on homogeneous servers. However, using homogeneous servers is not always pragmatic in a real-world IoT environment. The use of operating points enables a uniform description for the resources needed to serve a specific number of requests in heterogeneous servers. Therefore, future plans aim to conduct further research on resource elasticity, modifying the Central Controller in order to address the use case of heterogeneous servers. Finally, regarding the IDM component, it lies among the plans of the authors to evaluate further the accuracy of the proposed integrated decision making mechanism in support of fire detection based on real wildfire incidents and extend this to support the detection of other emergencies (e.g., floods or tsunamis).

## Figures and Tables

**Table 1:** Server operating points.

	TensorFlow	Conventional	TensorFlow	Infrared
Cores	X (s)	U (reqs)	X (s)	U (reqs)
1	3.0	4.2417	3.5	4.9487
2	3.0	14.2357	3.5	16.6083
3	3.0	17.1731	3.5	20.0353
4	3.0	18.4604	3.5	21.5371

**Table 2:** A review of wildfire incidents and related Tweets volumes.

Year	Country	Incident Location	Duration (Days)	Fire-Related Tweets	Reference
2012	USA	Colorado	32	4.2 K	[44]
2013	Australia	Australia	21	2.0 K	[44]
2014	Indonesia	Sumatra	92	9.7 K	[45]
2014	USA	San Marcos, Bernardo	9	1.3 K	[25]
2015	USA	California	52	1.9 K	[46]

## Abbreviations

The following abbreviations are used in this manuscript:

IoT	Internet of Things
UAVs	Unmanned Aerial Vehicles
CPS	Cyber-Physical System
CPSS	Cyber-Physical Social System
SMOKE	Scalable edge coMputing frameworK for Early fire detection
UGVs	Unmmaned Ground Vehicles
ETSI	European Telecommunications Standards Institute
QoS	Quality of Service
VM	Virtual Machine
IDM	Intelligent Decision Making
EFFIS	European Forest Fire Information System
MILP	Mixed Integer Linear Optimization Problem
MEC	Mobile Edge Computing
vUAV	virtual Unmanned Aerial Vehicles
